# From respiratory diseases to nervous system disorders: Unraveling the certified causes of influenza‐associated deaths in Poland from 2000 to 2019

**DOI:** 10.1111/irv.13214

**Published:** 2023-11-12

**Authors:** Marcin Piotr Walkowiak, Dariusz Walkowiak

**Affiliations:** ^1^ Department of Preventive Medicine Poznan University of Medical Sciences Poznań Poland; ^2^ Department of Organization and Management in Health Care Poznan University of Medical Sciences Poznań Poland

**Keywords:** biometry, cause of death, epidemiology, excess mortality, influenza, mortality

## Abstract

**Background:**

This study aims to accurately estimate influenza‐associated deaths in Poland and their certified cause of death, due to significant discrepancies between official numbers and expected impact.

**Methods:**

Excess influenza‐associated mortality in Poland from 2000 to 2019 was calculated using Seasonal‐Trend Decomposition Procedure based on LOESS (STL), which can detect non‐linear trends and non‐sinusoidal cycles. Excess mortality was then used as an explanatory variable in a model predicting monthly fluctuations of officially recorded causes of death from 2010 to 2019.

**Results:**

A total of 142,000 conservative estimates of influenza‐associated deaths were identified, representing 1.86% of overall mortality. Only 0.61% of influenza‐associated deaths were officially recorded as influenza. Nearly half of certified influenza deaths were attributed to the seasonal baseline mortality, potentially doubling estimated impact based solely on influenza peaks. Influenza‐associated deaths were frequently recorded as respiratory diseases (24.36%), with majority attributed to underlying conditions such as cardiovascular diseases (45.31%), cancer (9.06%), or diabetes (2.66%). Influenza‐associated deaths were more commonly certified as nervous system diseases (1.84%) or mental disorders (1.04%), rather than influenza itself. There was a noticeable impact of influenza on secondary infections, such as meningococcal and gastrointestinal infections.

**Conclusion:**

These findings highlight the importance of improved estimation for informing public health policy decisions.

## 1 INTRODUCTION

According to official statistics in Poland, the reported impact of influenza seems to be remarkably low, with an average of fewer than 80 deaths annually during the period of 2010–2019.^1^ This notably low figure cannot be attributed to successful vaccination campaigns, as the annual vaccination rate remained around 4% throughout the analyzed period. However, despite the low vaccination rates, the detection of relatively high levels of glutamin antibodies suggests the presence of potentially significant natural immunity.^2^

The COVID‐19 pandemic has stimulated increased attention to virus detection, leading to a modest improvement in the identification of influenza cases. However, a report by the State Sanitary Inspectorate, which covers the end of the analyzed period, provides a succinct summary of the situation. According to the report, during the period of December 23–31, 2019, there were 138,171 recorded cases of influenza‐like illnesses. Out of those cases, only two individuals were actually tested for influenza, and just one test turned out to be positive. There were zero reported deaths, though this result is unsurprising with just a single case.^3^

The lack of sufficient testing measures may also lead to an overestimation of influenza prevalence. In late November 2022, the deputy health minister made a strong claim that there were 100,000 reported influenza cases weekly in what was apparently an effort to promote influenza vaccination.^4^ While it is true that there was an unusually early onset of the influenza season during that period, the implicit assertion of nearly 100% influenza positivity rate among individuals with influenza‐like illnesses was an extraordinary estimate, as Sentinel's estimates for Europe in general at that time hovered around 14%.^5^ Nevertheless, when Polish government estimates for number of weekly influenza infection during early part of influenza season range from 1 to 100,000, there is a significant need to narrow these estimates down to more useful values from a public health perspective, especially concerning mortality.

There are two main techniques to estimate influenza‐associated mortality.^6^ The first technique involves death certification and laboratory diagnosis, which is known to lead to underdetection. An alternative approach within this technique is to use as a proxy deaths from upper respiratory infections, which are commonly secondary infections. This rough estimation yielded consistent annual estimates of influenza‐associated respiratory deaths at 1817^7^ and 1961^8^ for Poland. The second technique entails inferring influenza‐associated deaths through sudden increases in excess mortality observed during the influenza season. It is highly effective in case of sudden pandemic waves, though it is likely to underestimate during prolonged periods of elevated mortality. Beyond year‐to‐year variability, results and assumptions of both techniques are subject to debate and may vary depending on methodology.^9^, ^10^ Unfortunately, the absence of Poland from the Euromomo network precludes the acquisition of influenza‐associated mortality data from this source.^11^ Furthermore, Euromomo's assumption that annual mortality follows a sine‐like pattern may be perceived as a serious oversimplification, particularly given previous research demonstrating regional variations^12^ and temporal changes^13^ in the pattern of mortality. Due to the lack of studies conducted in Poland, studies conducted in neighboring countries such as Czechia^14^ or Hungary^15^ could be used as a reference. Additionally, there was a single study of Polish COVID‐19 excess deaths that incidentally estimated influenza deaths to oscillate around 9000 annually during 2010–2019 period.^16^ Studies on the official recording of influenza‐associated deaths are scarce, with the most recent comprehensive study on this topic covering data from the United States from 1997 to 2007^17^ and Canada from 1990 to 1999.^18^

The objective of this study is to provide a comprehensive and accurate estimation of influenza‐associated deaths in Poland and analyze how these deaths were officially reported. This research aims to delve into the mechanisms underlying the underreported mortality attributed to influenza, providing valuable insights into the actual recording practices and contributing factors leading to influenza‐related deaths. By examining the certified causes of death, we aim to gain a better understanding of the intricate relationship between influenza infections, the underlying health conditions of the vulnerable population, and the toll caused by secondary infections. Understanding the nuances of how influenza‐associated deaths are recorded and the underlying causes attributed to them is essential for the development of effective strategies aimed at reducing the impact of influenza on public health. It is our hope that this research will not only contribute to the scientific discourse but also foster informed decision‐making and public awareness, emphasizing the importance of preventive measures.

## 2 METHODS

### 2.1 Data source

Mortality data recorded on a weekly basis and segregated into two‐decade age groups were sourced from Eurostat.^19^ The starting period was 2000‐W01, based on data availability, while the end period was 2020‐W30, as later observations were heavily affected by COVID‐19 mortality. Thus, results for fall 1999/2000 would most likely be a slight underestimate since the 2 weeks preceding the starting period were unavailable. As there was no noticeable excess mortality in Poland attributable to the first wave of COVID‐19,^16^ it is possible to roughly estimate influenza excess mortality in fall 2019/2020. However, those results should not be treated as representative for annual influenza season. The exact moment of ending the dataset does not directly affect the estimates of influenza mortality, although it was desired to include it for the purpose of using the longest possible time series to estimate baseline.

We obtained data on monthly deaths subdivided into causes from Statistics Poland.^1^ Due to data availability, the starting period was January 2010, while the last included period in the model was December 2019, as later periods could have been affected directly by COVID‐19 or indirectly through higher scrutiny concerning infectious diseases.

### 2.2 Calculation

Estimating excess mortality that can be plausibly attributed to sudden waves of infectious diseases requires modeling baseline mortality with serious simplifying assumptions. Due to the distribution of sudden periods of elevated mortality diverging from linear regression assumptions, the typical approach involves either excluding them through mixed linear regression, applying natural logarithm to the dataset, or both. However, typical methods assume that the default annual mortality pattern follows a sine‐like function,^20^ which is only partially supported by observational data.

Given that relatively long time series were available, it was possible to derive the idealized pattern from the dataset of logarithmized weekly deaths. Seasonal variability was detected using STL (Seasonal‐Trend Decomposition Procedure based on LOESS).^21^ The underlying trend, which reflects demographic and health changes, was anticipated to change gradually over time. Therefore, a 3‐year smoothing window was applied to capture these slow changes. However, despite assuming a fixed cyclical pattern and employing robust fitting techniques, the annual cycle still exhibited overfitting to random noise. To address this, a Gaussian filter with a sigma value of 2.5 was subsequently used to smooth the annual cycle.

The obtained trend line and cyclical component were iteratively fitted in linear regression models to initially separate the observed mortality into two components: highly variable excess mortality and a stable underlying baseline. In the first step, a simple linear regression was used for fitting. However, in subsequent steps, excess observations were excluded from the fitting process. A threshold was employed, which was set at one hypothetical standard deviation calculated based on the median number of weekly deaths under assumption that they follow a Poisson distribution. Excess observations were given a weight of 0, while the remaining observations were assigned a weight of 1. This weighted regression process was repeated two times. After fitting, the baseline was further divided, using the fitted trend line as the division line, assuming the cyclical component reaches its minimum value throughout the year. This division allowed the decomposition of weekly observations into three components: excess mortality (highly variable “peaks” mainly composed of influenza‐associated and heatwave‐related deaths), seasonal baseline (“waves” exhibiting a regular annual mortality pattern characterized by a minimum value in late summer and a maximum value in late winter), and flat baseline (reflecting mortality that is least affected by temporal factors).

Previous research has indicated that the peak of the influenza season in Poland occurs between January and March,^22^ which was used to model monthly fluctuations in cause of death. As weekly mortality data were available, assuming that the influenza season lasted from late December to early April (W51–W15) allowed for a more precise estimation of overall influenza‐associated excess mortality. To align with the monthly format of the recorded causes of death, the weekly excess mortality data was converted accordingly. In the backward stepwise regression, the monthly recorded causes of death were used as the dependent variable. The explanatory variables considered excess deaths in January to March, excess deaths during the remaining months, monthly seasonal baseline, monthly flat baseline, time trend, and a constant term. The backward regression process involved iteratively eliminating explanatory variables lacking statistical significance until all remaining variables achieved *p* < 0.01.

The statistical analysis was performed using Python 3.10, utilizing the pandas 1.4.3 library for data processing and analysis. The statsmodels 1.13.5 library was used to calculate regression models. The STL module within the statsmodels.tsa.seasonal package was utilized for decomposition of the time series data into seasonal, trend, and residual components. The scipy.ndimage 1.9.3 library was used for smoothing of time series data using a Gaussian filter. Data visualizations were generated using the matplotlib.pyplot 3.7.1 and plotly.express 5.14.1 libraries.

## 3 RESULTS

**FIGURE 1:**
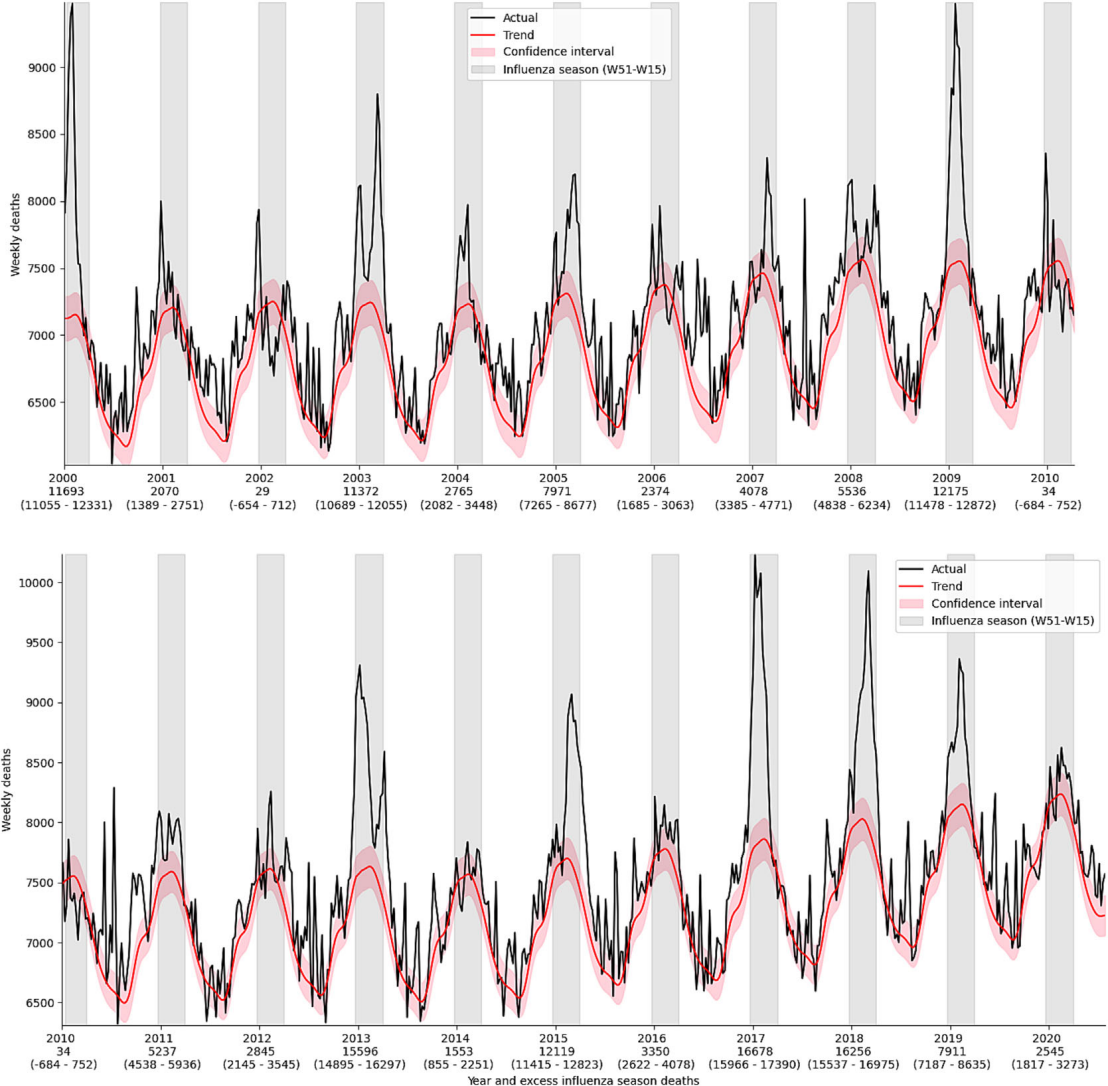
Polish weekly mortality in period 2000‐W01 to 2020‐W30.

The mortality in Poland during the analyzed period is presented in Figure 1, with the trend line created by the smoothed STL function showing some deviation from the typically used sine‐like function. Specifically, the model predicts an extended period of near‐minimal mortality rates during late summer preceding a sudden increase, which better fits the observational data. The rising trend in mortality rates was largely due to the aging of the population, compounded by the fact that the baby boomer generation is now approaching the age at which mortality rates begin to increase significantly.

The 95% confidence intervals are calculated based on the assumption that the baseline follows a Poisson distribution. These intervals effectively quantify the range of variability that can be attributed to random factors within the data. The largest peaks of mortality observed during the winter season are most likely attributable to influenza A, often having separate peaks for H3N2 and H1N1, as influenza B tends to cause smaller, later and harder to discern ones.^23^ The situation can be further affected by other viruses in the background. During the least deadly seasons of 2001/2002 and 2009/2010, there were clearly extended periods when mortality stayed below the baseline. The model captures influenza peaks well, although some of its epidemics have started or ended outside the analyzed bounds. There were on average 6867 (from 29 to 16,678) excess deaths due to influenza‐associated deaths annually. The lowest observations were well within the margin of error and should be understood as having no excess, while the overall distribution was positively skewed, with the median being only 5237. The overall number of influenza‐associated deaths in the 20‐year period from 2000‐W01 to 2019‐W52 was 144,187. When estimated separately in 20‐year age groups, the proportion of deaths attributable to influenza was 1.16% among those aged 0–19, 2.53% in the age group 20–39, 10.46% in the age group 40–59, 36.19% in the age group 60–79, and 49.67% in the age group 80 and above.

**FIGURE 2:**
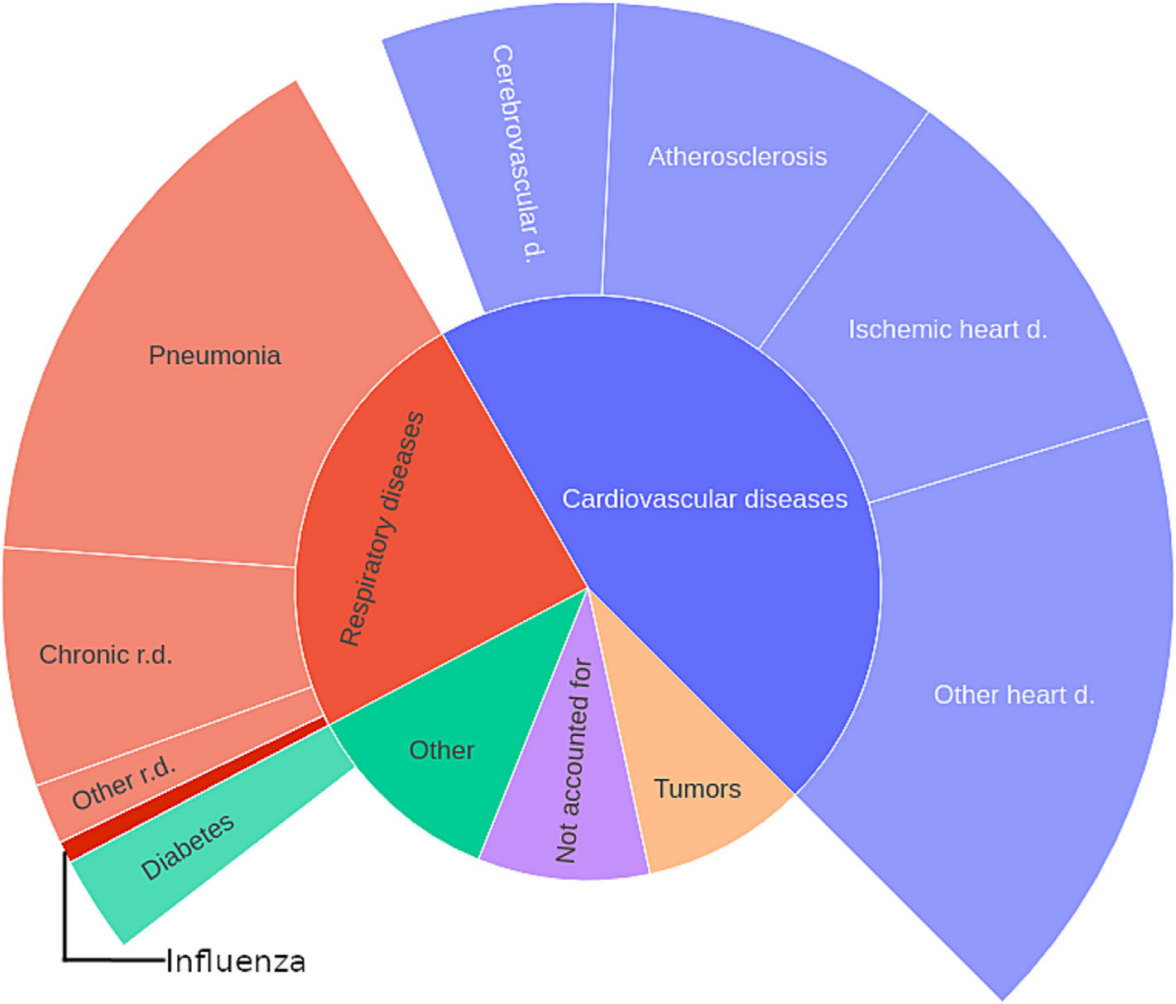
Official attribution of influenza‐associated deaths in years 2010–2019.

The main findings related to the attribution of 66,331 influenza‐associated deaths within the narrowed down range of January–March 2010–2019 are presented in Figure 2. Further detailed subdivisions and corresponding 95% confidence intervals are provided in Table 1. The subdivision applied in this study directly aligns with the classification used by Statistics Poland. Based on the model, the analysis revealed that 45.31% [C.I. 38.16%–52.47%] of influenza‐associated deaths were attributed to cardiovascular diseases. Interestingly, during the same period, cardiovascular diseases accounted for 43.80% of deaths in the general population. Notably, even when considering statistically significant subcategories, the attribution of influenza‐associated deaths closely resembled the underlying pattern observed for overall deaths. However, a slight deviation was noted for cerebrovascular diseases, where the observed prevalence of 8.14% slightly exceeded the upper bound of the estimated confidence intervals for influenza‐associated deaths. Despite the intricate nature of the underlying mechanisms, the level of consistency observed in these results implies that individuals with underlying cardiovascular diseases are highly prone to having their cause of death attributed precisely to the documented illness, without delving into the exact intricacies of the mechanism of their death.

**TABLE 1:** Official attribution of influenza‐associated deaths in years 2010–2019.

Officially recognized cause	Percentage of influenza excess deaths	95% confidence interval
Other infectious diseases	0.58%	[0.28%–0.89%]
Other gastrointestinal infections	0.28%	[0.18%–0.39%]
Meningococcal infection	0.03%	[0.02%–0.04%]
Tumors	9.06%	[6.29%–11.83%]
Malignant tumor of the lip, oral cavity, and pharynx	0.31%	[0.1%–0.52%]
Malignant tumor of the colon	0.71%	[0.28%–1.14%]
Malignant tumor of the trachea, bronchus, and lung	1.67%	[0.73%–2.61%]
Malignant melanoma of the skin	0.35%	[0.21%–0.49%]
Malignant tumor of the breast	0.97%	[0.69%–1.26%]
Malignant tumor of other and unspecified parts of the uterus	0.30%	[0.16%–0.44%]
Malignant tumor of the prostate gland	0.53%	[0.24%–0.82%]
Malignant tumor (other)	0.52%	[0.31%–0.72%]
Multiple myeloma and plasma cell tumors	0.38%	[0.24%–0.52%]
Leukemia	0.75%	[0.56%–0.94%]
Benign and unspecified tumors	0.74%	[0.24%–1.25%]
Endocrine disorders and nutritional deficiencies	2.92%	[2.35%–3.49%]
Diabetes	2.66%	[2.12%–3.21%]
Malnutrition	0.09%	[0.03%–0.14%]
Other endocrine disorders	0.16%	[0.07%–0.24%]
Mental disorders	1.04%	[0.55%–1.54%]
Non‐substance‐related mental disorders	0.32%	[0.18%–0.45%]
Neurological diseases	1.84%	[1.37%–2.31%]
Alzheimer's disease	0.94%	[0.68%–1.20%]
Other neurological diseases	0.88%	[0.55%–1.21%]
Cardiovascular diseases	45.31%	[38.16%–52.47%]
Ischemic heart disease	10.24%	[6.83%–13.65%]
Other heart diseases	17.17%	[8.9%–25.43%]
Cerebrovascular diseases	6.32%	[4.93%–7.70%]
Atherosclerosis	9.06%	[5.33%–12.79%]
Respiratory diseases	24.28%	[22.57%–26.00%]
Influenza	0.61%	[0.46%–0.77%]
Pneumonia	15.52%	[14.28%–16.75%]
Other acute lower respiratory infections	0.12%	[0.09%–0.15%]
Chronic respiratory diseases	6.53%	[5.77%–7.29%]
Other respiratory diseases	1.66%	[1.07%–2.25%]
Digestive system disorders	1.22%	[0.34%–2.10%]
Musculoskeletal disorders	0.22%	[0.13%–0.31%]
Urinary system disorders	1.56%	[0.77%–2.35%]
Glomerular diseases	0.78%	[0.43%–1.13%]
Falls	0.63%	[0.26%–1.00%]

Influenza‐associated deaths were classified as caused by respiratory diseases in 24.28% [C.I. 22.57%–26.00%] while respiratory diseases were responsible only for 5.91% deaths in general. However, only 0.61% [C.I. 0.46%–0.77%] of influenza‐associated deaths was directly attributed to influenza. While this classification may seem somewhat misleading from an epidemiological standpoint, it is likely a highly accurate description of secondary infections that follow influenza. The only subcategory that appeared rather misclassified was chronic respiratory diseases, which accounted for 6.53% [C.I. 5.77%–7.29%] of these deaths. However, the model provided another worrisome insight—it attributed influenza deaths not only to periods of influenza excess deaths but also to the seasonal component. This finding, in itself, might not be particularly surprising, given that the assumed influenza season (W50–W15) had to be truncated to January–March in this model to align with the available monthly observations. Nevertheless, the scale of this attribution was staggering, as the total number of influenza deaths in the seasonal component was even greater than during the analyzed peaks, albeit not statistically significant.

Influenza‐associated deaths were attributed to tumors in 9.06% [C.I. 6.29%–11.83%] of cases, while tumors accounted for 26.24% of overall deaths during the same period. A clear pattern emerges from the data. The attribution of influenza‐associated deaths does not align with poorly prognostic cancers such as pancreatic or liver cancer. Instead, it is predominantly associated with tumors that have more favorable prognoses, such as, for example, those affecting the prostate gland or tumors that were not diagnosed as malignant, suggesting incidental findings. However, there may also be a genuine link between influenza and tumors, notably with malignant tumors of the trachea, the bronchus, and the lung, which generally have a poor prognosis. In this case, the presence of the tumor may contribute to a higher susceptibility to subsequent infections, leading to increased mortality.

Based on the model, there is a wide range of underlying conditions that contribute to increased vulnerability and are ultimately listed as the official cause of death. A percentage of 2.92% [C.I. 2.35%–3.49%] is attributed to endocrine disorders and nutritional deficiencies, predominantly diabetes. Similarly, attributing deaths to digestive system disorders, urinary system disorders, or glomerular diseases seems plausible as these conditions can make individuals more vulnerable to adverse outcomes. However, some categories appear less plausible as primary causes of death, as they include musculoskeletal disorders, neurological diseases, and even mental disorders. Surprisingly, these categories were more likely to be officially attributed than influenza. Notably, one category was missing from the model's findings, providing insight into the rationale behind attribution. Although the model detected categories responsible for less than 1% of deaths, it did not find any association between influenza‐associated deaths and the category accounting for 7.20% of deaths during the studied period, namely, symptoms, signs, and abnormal clinical and laboratory findings. The data suggest a potential tendency to attribute deaths to clear underlying conditions without extensive examination, while inadequate known conditions prompted deeper investigation into recent infections.

While the overall attribution of influenza‐associated deaths to other infectious diseases was relatively small at 0.58% [C.I. 0.28%–0.89%], it is worth noting that influenza appears to have a discernible impact on secondary infections beyond those typically affecting the respiratory tract. Deaths were also attributed to other gastrointestinal infections (0.28% [C.I. 0.18%–0.39%]) and even meningococcal infection (0.03% [C.I. 0.02%–0.04%]).

## 4 DISCUSSION

Despite significant advancements in medical diagnosis and treatment, the findings of this study are highly consistent with much earlier research. As early as 1932, Collins reported that “46 per cent of such excess deaths were credited to organic heart disease,”^24^ which incidentally a century later perfectly matches our results. Similar impact on cardiovascular deaths was observed, apparently independently by others like, for example, Eickhoff et al. in 1961.^25^ However, recent studies specifically covering influenza‐associated mortality in Poland have focused solely on respiratory mortality. In the case of Paget et al.,^8^ it was concluded that including cardiovascular deaths would at least double their estimated death toll, while in the case of Iuliano et al.,^7^ this key limitation was not even addressed. The approach of covering only respiratory diseases responsible for 24.28% [C.I. 22.57%–26.00%] of influenza‐associated mortality, while ignoring cardiovascular diseases 45.31% [C.I. 38.16%–52.47%], seems deeply flawed.

Despite comparing Polish results with those obtained in Canada and the United States, the general patterns remained consistent. Influenza‐associated deaths were primarily attributed to cardiovascular diseases, with respiratory diseases as the second most common cause, but among them, only a small fraction was actually attributed to influenza. On the third place were cancers, and further down the list, it was possible to track some minor causes. Goldstein et al. employed less specific categorization for their study on influenza‐associated deaths in the United States between 1997 and 2007. Nevertheless, their findings matched ours in identifying less intuitive categories such as Alzheimer's disease, renal disease, or diabetes as attributed causes of influenza‐associated deaths.^17^ Results from Schanzer et al. for Canada in the years 1990–1999^18^ were highly similar; for example, they attributed 0.9% of deaths to psychotic conditions, while our sample indicated 1% was attributed to mental disorders. While it may appear to be an accidental finding, mental illnesses have been identified as an independent risk factor for COVID‐19 deaths; thus, the nature of this relationship remains unclear.^26^ It is worth noting that in their model, some influenza‐associated deaths were attributed to external causes, which they dismissed as spurious correlation. For example, 0.9% of deaths were attributed to falls, while in our model, this odd explanation was responsible for 0.6% of deaths in our sample. This raises the possibility that these were not pure statistical anomalies in both cases but rather genuine mechanisms, such as low temperature increasing likelihood of falls and modulating the timing of influenza‐associated deaths or alternatively influenza‐associated deaths occurring outside of beds being attributed to falls.

From a practical perspective, there are three primary conclusions. Firstly, there is a need to enhance the quality of reporting deaths associated with viral infections. This is exemplified as waves of mysterious “sudden cardiovascular death” become a subject warranting investigation within forensic journals, though their core suspect turns out to be “cold and dry weather”.^27^ However, even when correctly identifying the problem, the issue remains challenging as for the 2017/2018 season, Germany confirmed only 1,674 of the indirectly estimated 25,100 influenza‐associated deaths.^28^ Remarkably, no deaths were attribute to HIV in Poland over a span of 10 years.^1^ Similarly, during the second wave of COVID‐19, only about half of the excess deaths were officially attributed to COVID‐19.^1^ However, reporting of deaths has improved since the third wave^16^ though issues with reporting infections remained^29^ indicating partial progress in addressing the issue. The approach of separately reporting deaths where viral infections are at least one of the underlying causes, as done for COVID‐19, seems suitable.

The second recommendation is related to improving prevention efforts and accurately attributing deaths to influenza in patients with underlying health conditions, who were found to be at high risk for influenza‐associated mortality based on the model. Fleming et al. observed a paradox whereby there was no increase in patient visits to general practitioners or emergency rooms for cardiovascular diseases during influenza pandemics, yet there was a spike in cardiovascular deaths.^30^ Moa et al. found some increase in hospitalizations for cardiovascular diseases during influenza pandemics, but it was statistically significant only for the age group 65 and above. Even in this group, the increase was approximately one additional cardiovascular hospitalization for every six additional respiratory hospitalizations.^31^ While the high mortality rate combined with a low number of less severe cases raises questions concerning attribution, more importantly, studies on patients with severe cardiovascular conditions show that influenza vaccination can significantly improve their prognosis^32^, ^33^

Studies on multiple unrelated conditions have demonstrated a similar pattern, with barely noticeable seasonal variability in amputation rates for critical limb‐threatening ischemia, but hospitalization patterns aligning with influenza waves.^34^ An elevated incidence of hospitalization for acute kidney injury was observed in winter, and during this period, the mortality rate among those hospitalized was found to be highest.^35^ An increased winter mortality rate was observed in intensive care units, which remained statistically significant even after adjusting for diagnosis.^36^ Common pattern emerges among these studies—little increase in typical symptoms, some increase in hospitalization, and significant increase in mortality. In some studies, influenza was not even openly mentioned, which is consistent with symptoms not matching and only suspicious timing suggestively implicating the virus.

The third recommendation pertains to less obvious secondary infections. According to the model, 14.53% [C.I. 8.76%–20.29%] of meningococcal deaths were attributed to influenza‐associated deaths, which is consistent with the established links between influenza and secondary meningococcal infections.^37^, ^38^ Targeting any part of the infection chain is possible, as Goldstein et al. had to adjust their model after the introduction of pneumococcal conjugate vaccination resulted in a decrease in this particular type of influenza‐associated deaths.^17^ Nevertheless, this study shows significant gains from prioritizing influenza, as it can lead to modest gains in relation to multiple conditions.

### 4.1 Limitations

Additional caution should be exercised when attempting to extrapolate our findings. First, Polish society is aging, and as a result, the overall number of deaths is expected to increase. Second, COVID‐19 restrictions initially suppressed viruses in general, giving rise to an unusually large and untimely resurgence in 2022.^39^ Therefore, these resuls are unlikely to be representative in the immediate aftermath of the pandemic. To calculate influenza‐associated mortality, this model assumes a constant baseline with random variability following a Poisson distribution, unless influenced by external factors like meteorological and epidemic conditions. However, other studies have employed two different approaches, potentially leading to diverging conclusions. Some studies have prioritized the observation of overdispersion,^37^ resulting in the adoption of negative binomial models, while others have focused on identifying negative autocorrelation, leading to the use of ARIMA‐derived models. Nevertheless, addressing this issue by modeling overdispersion would require estimating the baseline overdispersion based on overdispersion primarily caused by influenza waves and heatwaves. Moreover, there is no compelling reason to assume that these two distributions are identical. Furthermore, it would need to be further corrected for negative autocorrelation, which is not even constant and becomes more visible in the aftermath of heatwaves,^40^ though influenza deaths were noticed to lead to reduced heatwave deaths in subsequent summer.^41^ Apart from the assumption‐heavy calculations, treating this as random overdispersion disregards the underlying mechanism. Instead, we consider it to be a non‐random mechanism that could introduce difficult‐to‐quantify bias, especially under highly unusual conditions such as different weather patterns or the emergence of significant viruses (as observed with COVID‐19; thus, our dataset ends at 2019).

## AUTHOR CONTRIBUTIONS

Marcin Piotr Walkowiak and Dariusz Walkowiak conceived and designed the study. Marcin Piotr Walkowiak obtained and managed the data. Marcin Piotr Walkowiak and Dariusz Walkowiak developed the analytical strategy. Marcin Piotr Walkowiak conducted analysis, interpreted the data, and drafted the figures. Marcin Piotr Walkowiak wrote the first draft of the manuscript. All authors provided input to finalize the paper. Marcin Piotr Walkowiak and Dariusz Walkowiak had full access to all data used in the study. All authors were responsible for submitting the article for publication.

## CONFLICT OF INTEREST STATEMENT

We declare no competing interests.

### PEER REVIEW

The peer review history for this article is available at https://publons.com/publon/10.1111/irv.13214.

## Data Availability

All used data are publicly available.
